# Supplementary material for: Causal Relationship between Aging and Anorexia Nervosa: A White-Matter-Microstructure-Mediated Mendelian Randomization Analysis
Source: Biomedicines. 2024 Aug 16;12(8):1874. doi: 10.3390/biomedicines12081874 (PMC11351342; doi:10.3390/biomedicines12081874)
Supplement: Supplementary file 1 [file biomedicines-12-01874-s001.zip › Supplementary Figure/Figure S9/Leaveoneout plot of MR analysis for ubm-b-1928 to AN.pdf]

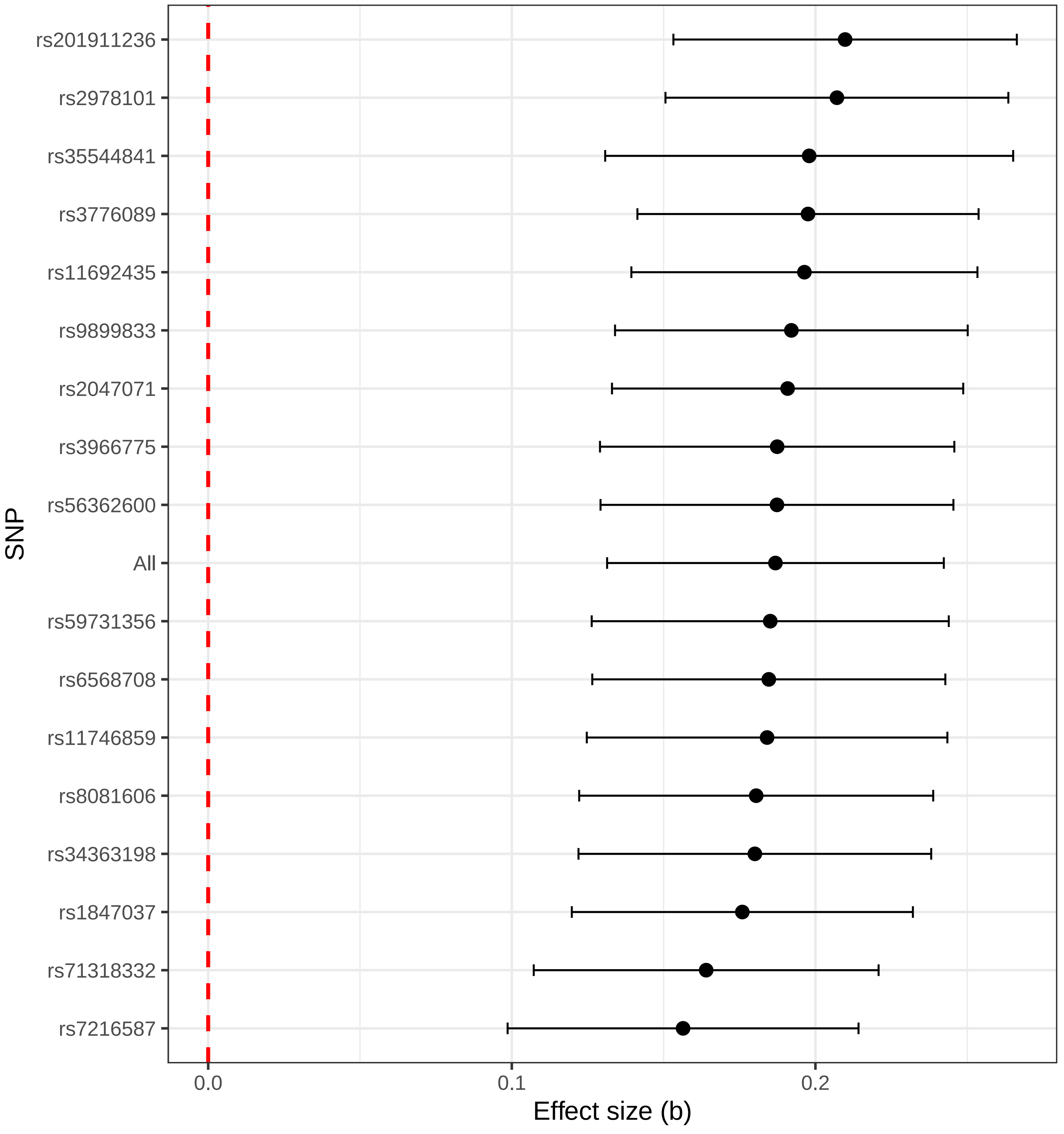

rs201911236
rs2978101
rs35544841
rs3776089
rs11692435
rs9899833
rs2047071
rs3966775
rs56362600
All
rs59731356
rs6568708
rs11746859
rs8081606
rs34363198
rs1847037
rs71318332
rs7216587
SNP
0.0
0.1
0.2
Effect size (b)
